# Renal hypouricemia type 2 with SLC2A9 compound heterozygous variants: a case report of recurrent acute kidney injury triggered by low-intensity exercise

**DOI:** 10.3389/fneph.2024.1463913

**Published:** 2024-10-03

**Authors:** Niranjana Rekha Paladugu, Muralinath Vukkadala

**Affiliations:** Department of Nephrology, AIG (Mayo Clinic Care Network) Hospitals, Hyderabad, Telangana, India

**Keywords:** renal hypouricemia, exercise-induced acute kidney injury, SLC2A9 gene variants, fractional excretion of uric acid, compound heterozygous

## Abstract

Renal hypouricemia (RHUC) is a rare genetic disorder characterized by impaired uric acid reabsorption which leads to persistently low serum uric acid levels. This condition predisposes individuals to complications such as uric acid kidney stones and exercise-induced acute kidney injury (EIAKI). Although mutations in SLC22A12 and SLC2A9 are commonly implicated in RHUC, the precise pathophysiological mechanisms, particularly those contributing to AKI, remain incompletely understood. We report the case of a 30-year-old male who experienced recurrent episodes of EIAKI despite the absence of high-intensity exercise, suggesting the involvement of factors beyond the traditional risk. Genetic analysis confirmed the diagnosis of RHUC type 2 (RHUC2) and identified compound heterozygous variants of SLC2A9. Although these variants are not novel, this case contributes to the limited literature on RHUC2, particularly in male patients with recurrent EIAKI. These findings highlight the importance of maintaining a high index of suspicion for RHUC in cases of unexplained AKI, especially when recurrent episodes follow physical activity, and the need for targeted genetic testing for an accurate diagnosis. The genomic data related to this case are available in Mendeley Data: Vukkadala, Muralinath; Paladugu, Niranjana Rekha (2024), “Renal hypouricemia,” Mendeley Data, V2, doi: 10.17632/7z84mkdgn9.2.

## Introduction

Renal hypouricemia (RHUC) is a rare genetic disorder marked by defective uric acid reabsorption in the kidneys, resulting in persistently low serum uric acid levels. This condition increases the risk of uric acid kidney stones and exercise-induced acute kidney injury (EIAKI), especially post-exertion. RHUC is categorized into RHUC type 1 (RHUC1) and RHUC type 2 (RHUC2) based on genetic mutations. RHUC1 is linked to mutations in SLC22A12, which encodes the URAT1 transporter essential for uric acid reabsorption. RHUC2 is associated with mutations in SLC2A9, encoding the GLUT9 transporter, also critical for uric acid reabsorption. Both types lead to hypouricemia and related complications (1).

Recent studies have pinpointed specific genetic mutations causing RHUC, such as those in SLC22A12 and SLC2A9, but the exact mechanisms by which these mutations contribute to RHUC-related AKI, including oxidative stress and renal vasoconstriction, remain unclear. Aomura et al. (2020) indicated that although some RHUC patients develop EIAKI at varying levels of physical exertion, additional risk factors likely play a role in the condition’s onset beyond exercise alone (2). This underscores the necessity for a thorough investigation into the interplay between genetic predispositions and other potential risk factors for EIAKI development.

We present a case of a 30-year-old Indian male experiencing recurrent post-exercise AKI without prior high-intensity exercise. Whole-genome sequencing confirmed RHUC2 diagnosis by identifying compound heterozygous variants in SLC2A9. These findings emphasize SLC2A9 variants’ role in EIAKI pathogenesis and raise considerations about sex-based susceptibility. Although these variants are not novel, this case enhances the understanding of RHUC2, especially regarding variants of uncertain significance (VUS), and adds to the syndrome’s genetic diversity. The genomic data have been published in Mendeley Data to support further research and validation (Vukkadala, Muralinath; Paladugu, Niranjana Rekha (2024), “Renal hypouricemia,” Mendeley Data, V2, doi: 10.17632/7z84mkdgn9.2).

## Case report

In June 2023, a 30-year-old male presented with a three-year history of recurrent, self-resolving acute kidney injury (AKI), exhibiting symptoms of nausea, generalized weakness, and occasional hematuria. His medical history revealed no chronic conditions, family history of kidney disease, smoking, alcohol use, NSAIDs, or other medications. The patient had previously visited multiple hospitals and consulted various physicians.

### Clinical history

The patient’s health was stable until November 2020, when he sought medical attention for nausea and weakness, with a serum creatinine level of 2 mg/dL. He was treated with intravenous hydration (1000 ml/day) for three days, reducing serum creatinine from 2.0 mg/dL to 1.1 mg/dL without additional pharmacological intervention. In April 2021, he contracted COVID-19, with a creatinine level of 1.4 mg/dL, recovering within five days of conservative management. In September 2021, he experienced similar symptoms of nausea and weakness, with a creatinine level of 1.5 mg/dL. A complete urine examination yielded negative results, with a urine protein-to-creatinine ratio of 0.04. Laboratory tests, including complete blood counts, liver function tests, and iron levels, were normal. During this period, the patient had been using Ayurvedic medicine for one month.

In November 2021, the patient experienced nausea and generalized weakness with a creatinine level of 1.6 mg/dL. Urine examination showed 14-16 RBCs/HPF, but no albumin or WBCs. The 24-hour urine protein was 72 mg, and the ANA profile was negative. A renal biopsy was suggested but postponed. A month later, the creatinine level normalized to 1.1 mg/dL, and the urine examination was negative.

In May 2022, the patient had gross hematuria, and creatinine was 2.57 mg/dL. Urine analysis showed RBCs and WBCs (5-6 cells/HPF). The ASO titer was negative, and C3 and C4 levels were normal. Suspected acute interstitial nephritis was treated with intravenous solumedrol 500 mg for three days, reducing creatinine to 1.7 mg/dL, and stabilizing at 1.2 mg/dL within a week. Subsequent urine examination revealed no abnormalities. A one-month tapering course of oral steroids was prescribed.

In June 2022, the patient had similar symptoms with a creatinine level of 2.7 mg/dL. Urine examination was negative. A renal biopsy showed unremarkable glomeruli, tubulointerstitial compartments, and vessels. Electron microscopy showed normal glomerular basement membrane thickness and well-preserved visceral epithelial cell foot processes. Within a week, creatinine decreased to 1.4 mg/dL. Blood examination, including hemoglobin, hematocrit, and RBC indices, was normal, ruling out anemia. Liver function tests and iron levels were also normal, excluding iron deficiency.

The patient maintained a daily exercise regimen of 60 minutes, including aerobic activities and moderate strength training with both isotonic and isometric movements, without using protein supplements. The diet included a moderate amount of meat. Family history was unremarkable for kidney diseases or genetic conditions. Psychosocial history, including lifestyle and stress levels, did not reveal significant contributing factors.

### Clinical evaluation and genetic diagnosis

The patient was well until June 2023 when he experienced prodromal symptoms and significant hematuria. Serum creatinine was 2.3 mg/dL. Urine examination showed numerous RBCs and a negative WBC count. Total bilirubin was 1.64 mg/dL, direct bilirubin 0.25 mg/dL, and indirect bilirubin 1.39 mg/dL. Imaging studies, including ultrasound and CT, showed no kidney stones or structural abnormalities.

Despite no significant glomerular or tubular damage on kidney biopsy and a normal glomerular basement membrane, recurrent AKI episodes with hematuria suggested exercise-induced acute kidney injury. Laboratory tests indicated elevated creatine phosphokinase (CPK) levels at 460 U/L, three times the normal level, while lactate dehydrogenase (LDH) remained normal. Calcium, phosphate, bicarbonate, and intact parathyroid hormone (iPTH) levels were normal. Uric acid was low at 0.3 mg/dL, with a fractional excretion of uric acid at 120% (normal, <10%). Creatinine normalized to 1.1 mg/dL within a week.

**Figure 1:**
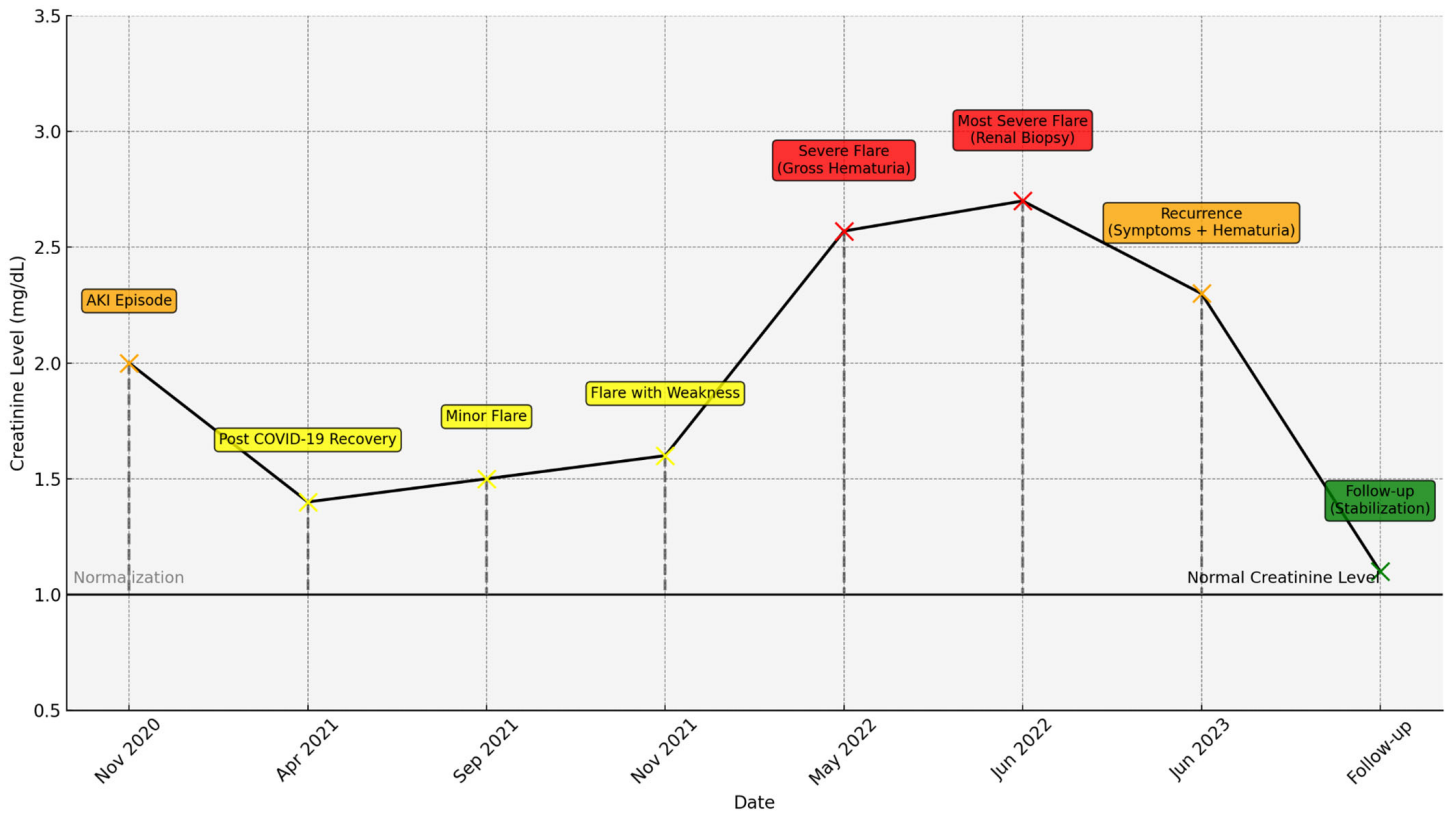
Creatinine levels over time with key clinical events, color-coded (yellow for mild, orange for moderate, red for severe), and dashed lines indicating normalization periods.

Throughout the follow-up period, the patient did not exhibit any signs of hypertension, with blood pressure consistently within the normal range, and no hypertensive episodes were observed during the treatment (**Figure 1**).

**Figure 2:**
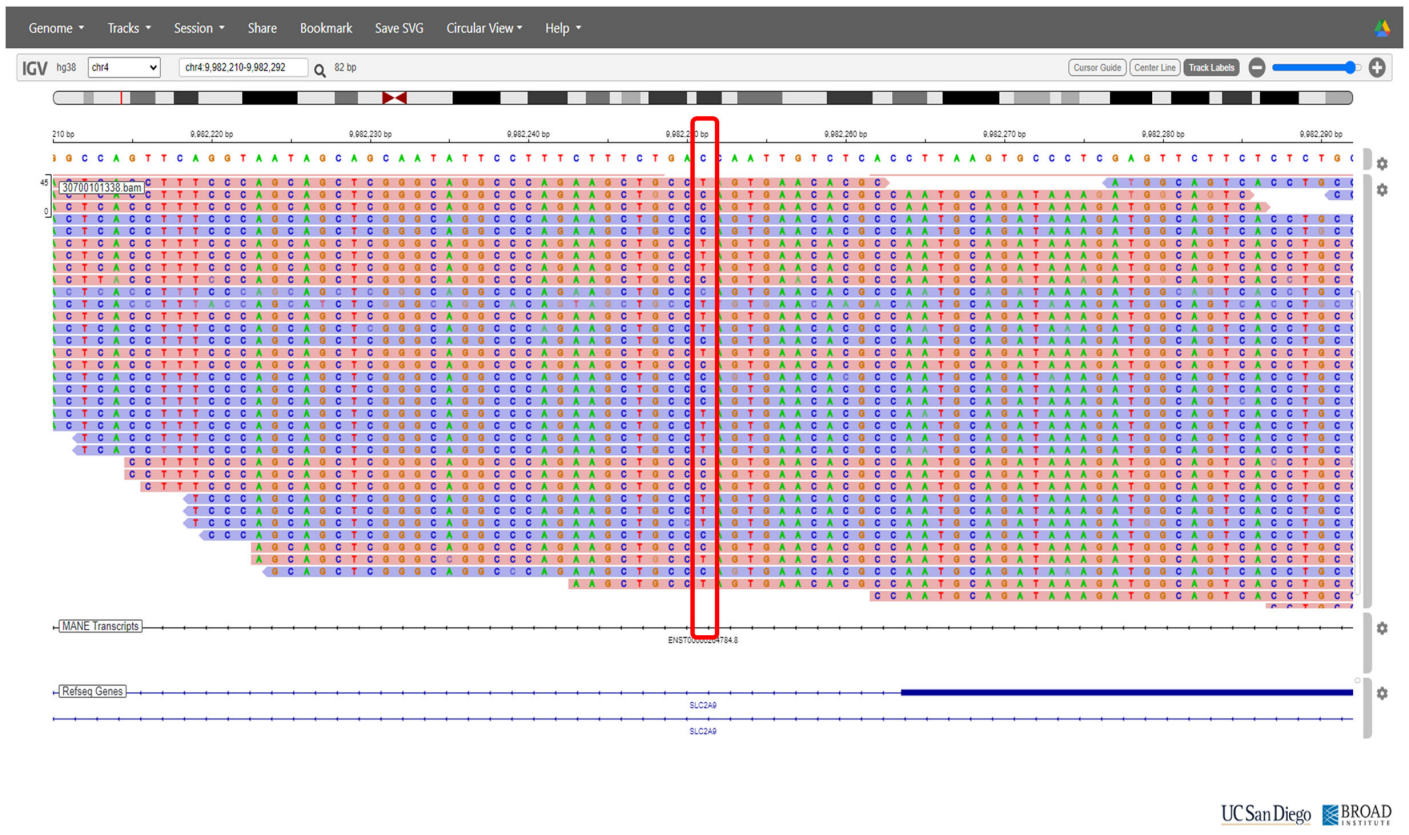
Chromosomal position of significant variant c.646G>A in the SLC2A9 gene.

**Figure 3:**
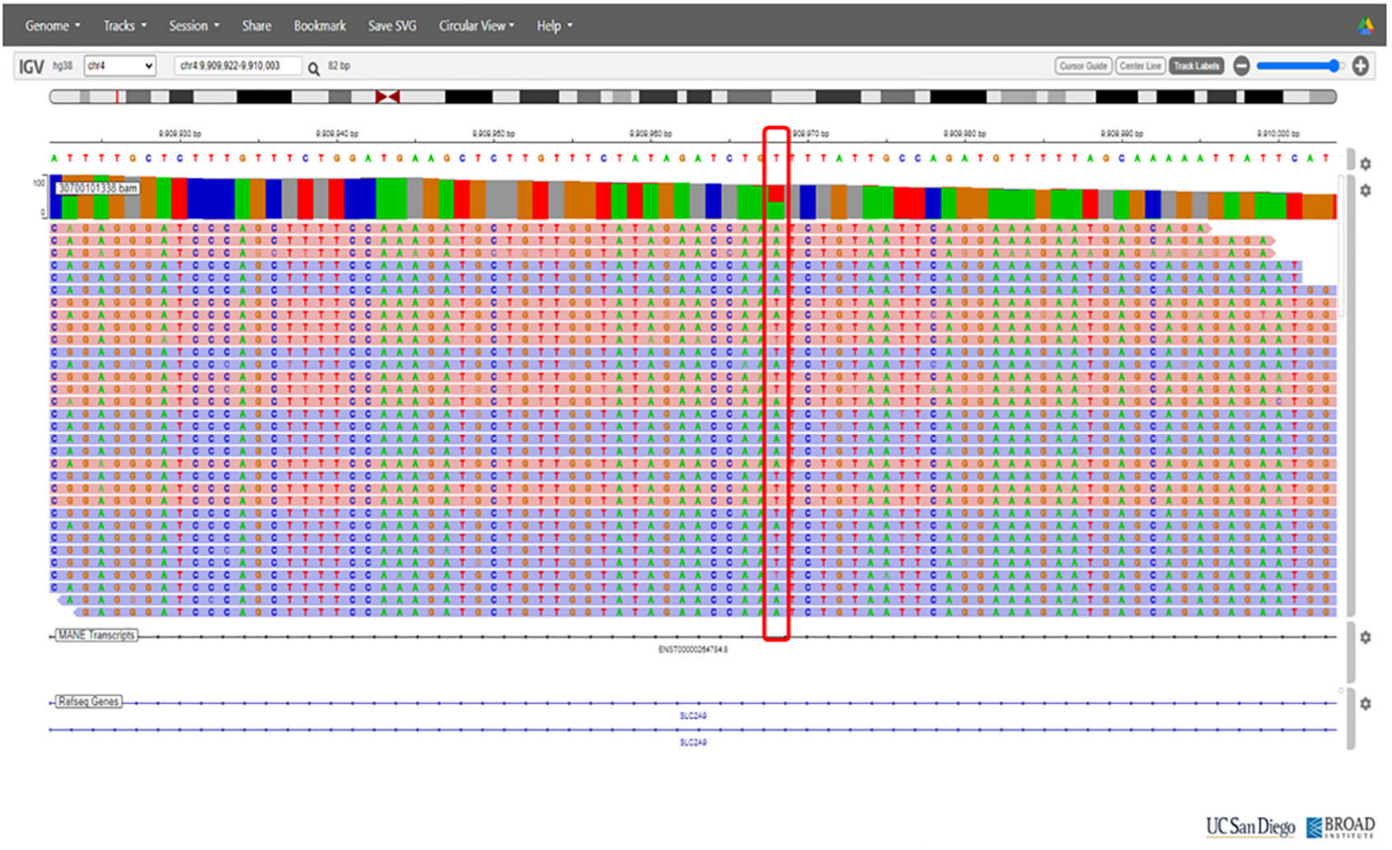
Chromosomal position of significant variant c.1004T>A in the SLC2A9 g.

**Figure 4:**
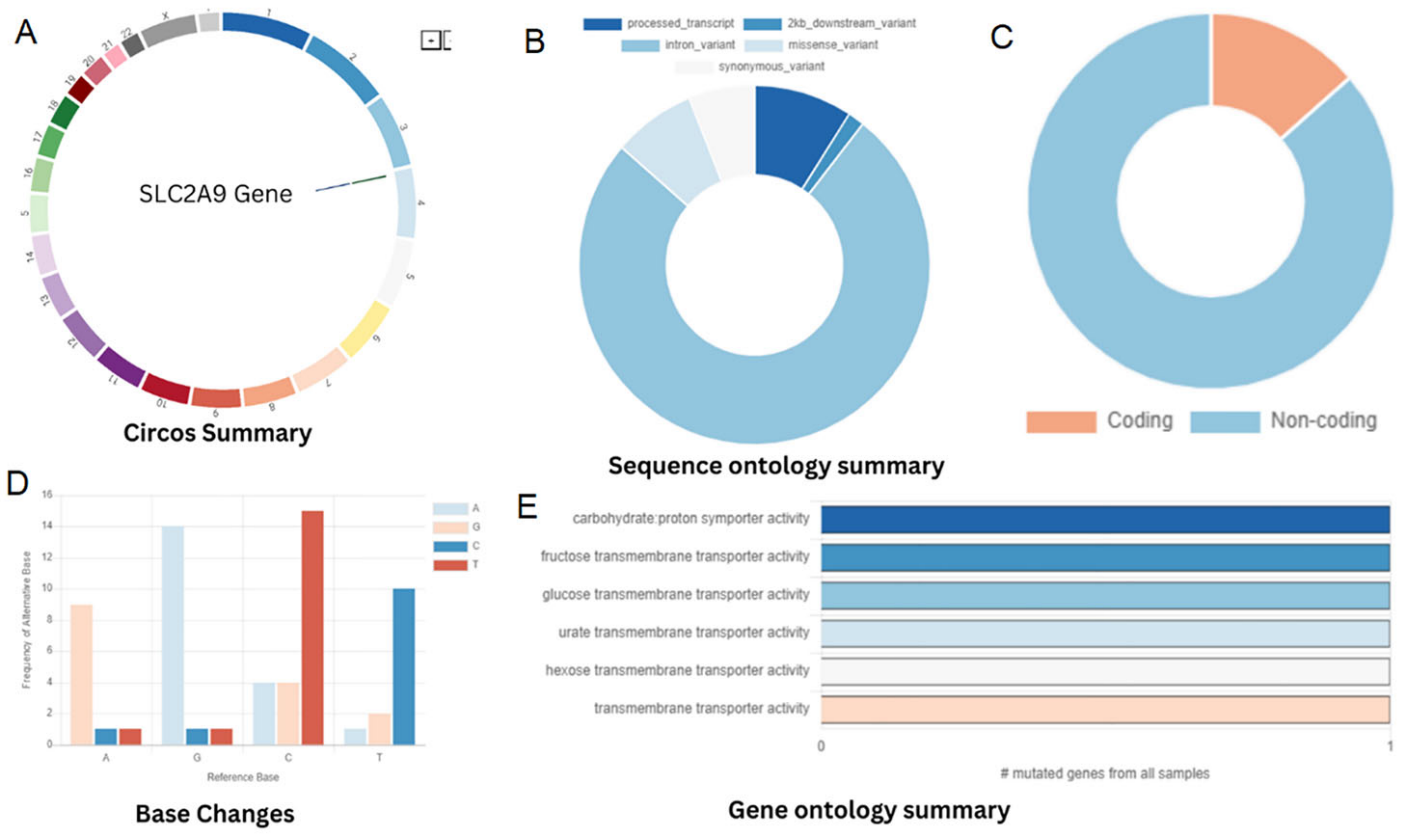
**(A)** Chromosomal location of SLC2A9 gene. **(B)** Classification of SNPs. **(C)** Percentage of genes distributed among the protein-coding and non-coding genes. **(D)** Base alteration frequency. **(E)** Gene ontology.

On the basis of the clinical presentation, renal hypouricemia was suspected. Whole-genome sequencing (WGS) confirmed the diagnosis of RHUC type 2 by identifying compound heterozygous mutations in SLC2A9: c.646G>A in exon 5 and c.1004T>A in exon 8 (**Figures 2** and **3**, respectively; **Figure 4**). These findings were consistent with the diagnosis of RHUC2. The family members were screened for uric acid levels, which were normal at the time, and genetic testing was recommended but they denied.

### Management and follow-up

The patient was provided with comprehensive information regarding RHUC2 and prognosis. The patient received supportive care, which included strict monitoring of fluid intake to maintain adequate hydration, correction of electrolyte imbalances with oral supplements, and close observation for any signs of worsening kidney function, adhere to a low-purine diet, and modify his exercise routine to include non-aggressive activities, such as walking, light jogging, swimming, or moderate cycling. Allopurinol has been discussed as a potential treatment option for acute episodes; however, given the lack of robust evidence, it has not yet been prescribed. During the two-month follow-up, the patient’s creatinine levels remained stable at 1.1 mg/dL, with no further episodes of AKI.

## Discussion

### Overview of renal hypouricemia

Renal Hypouricemia (RHUC) is a rare genetic disorder marked by impaired uric acid reabsorption in the kidneys, leading to persistently low serum uric acid levels. While many individuals with RHUC are asymptomatic, the condition can cause significant complications, including uric acid kidney stones and exercise-induced acute kidney injury (EIAKI). The rare and often silent nature of RHUC presents diagnostic challenges, necessitating a comprehensive understanding of its mechanisms and clinical implications for effective management (3).

### Genetic basis and the role of SLC2A9 and GLUT9 transporters

RHUC pathogenesis centers on the dysfunction of the GLUT9 transporter, encoded by the SLC2A9 gene. This transporter is crucial for uric acid reabsorption in the renal proximal tubules. Mutations in the SLC2A9 gene disrupt GLUT9 function, decreasing reabsorption and increasing urinary excretion of uric acid (4). This dysfunction leads to hypouricemia and a higher risk of kidney stones and EIAKI. Understanding the GLUT9 transporter and the impact of SLC2A9 mutations is essential for recognizing and managing RHUC’s clinical manifestations.

### Pathophysiological mechanisms in RHUC

The pathophysiology of RHUC involves genetic mutations, oxidative stress, and renal hemodynamic changes, which are essential for understanding related complications.

Impaired Uric Acid Reabsorption: Mutations in the SLC2A9 gene result in defective GLUT9 transporters, reducing uric acid reabsorption from renal tubules. This leads to low serum uric acid levels and increased urinary excretion, contributing to uric acid crystals and kidney stones.

Exercise-Induced Acute Kidney Injury (EIAKI): Physical exertion raises the demand for uric acid clearance. In RHUC individuals, defective GLUT9 transporters fail to manage this increased load, causing uric acid accumulation in renal tubules. This triggers oxidative stress, inflammation, and renal vasoconstriction, key factors in AKI development (5).

Oxidative Stress and Inflammation: Uric acid accumulation in renal tubular cells generates reactive oxygen species (ROS), leading to oxidative stress. This, along with inflammation, damages renal tissues and increases AKI risk (6).

Renal Vasoconstriction: Physical activity can cause transient renal vasoconstriction and reduced renal blood flow. In RHUC, where uric acid reabsorption is already impaired, this further reduction in renal perfusion heightens the risk of ischemic injury (7).

### Genetic testing in RHUC

Genetic testing is vital for confirming RHUC diagnosis and distinguishing between RHUC types 1 and 2. RHUC1 involves mutations in SLC22A12, responsible for the URAT1 transporter, while RHUC2 involves mutations in SLC2A9, which encodes the GLUT9 transporter. Identifying these mutations confirms the diagnosis and provides insights into inheritance patterns and potential risks for family members. Advanced methods like whole-genome sequencing (WGS) and whole-exome sequencing (WES) are effective in identifying these mutations, especially when clinical presentation is unclear or rare variants are suspected (8). However, due to their complexity and cost, targeted genetic panels focusing on commonly implicated RHUC genes are often preferred for efficient and accurate diagnosis.

### Clinical presentation, diagnosis, and management of RHUC

Diagnosing RHUC, especially when leading to EIAKI, requires a high index of suspicion due to the silent nature of the condition and non-specific AKI symptoms like fatigue, nausea, and weakness. A thorough clinical history is essential, particularly in patients with AKI following physical exertion. Important history aspects include previous AKI episodes, kidney stones, and the intensity and duration of physical activity preceding symptoms.

### Diagnostic evaluation

- Hypouricemia, defined as serum uric acid < 2 mg/dL, is a key indicator but not diagnostic alone, as low uric acid can occur in various conditions.
- Evaluating urine uric acid levels and fractional excretion of uric acid (FEUA) is crucial for diagnosis. High urinary uric acid with low serum levels indicates a renal reabsorption defect characteristic of RHUC (9).

### Management of RHUC-AKI

- Supportive Care: Prioritize hydration, correct electrolyte imbalances, and monitor for early AKI signs.
- Pharmacological Intervention: Xanthine oxidase inhibitors like allopurinol might reduce uric acid production, though evidence for this in RHUC is limited.
- Patient Education: Advise on lifestyle changes, such as avoiding high-purine foods and nephrotoxic medications, to prevent RHUC-AKI recurrence (8).

### Insights from this case and comparison with other studies

This case of a 30-year-old Indian male with RHUC and EIAKI reveals the genetic and clinical complexities of RHUC, particularly with compound heterozygous mutations in the SLC2A9 gene. It enhances understanding of genetic variations and their clinical manifestations, and how these variations compare with similar cases reported in the literature.

### Genetic insights and variability

The compound heterozygous mutations in the SLC2A9 gene, c.646G>A (p.Gly212Arg) and c.1004T>A (p.Ile335Asn), demonstrate the genetic diversity linked to RHUC. The I335N variant, classified as a Variant of Uncertain Significance (VUS), is especially significant. Its relatively higher frequency in South Asian populations (0.036% in gnomAD) and its intron-exon boundary location suggest potential splicing impact, although its clinical significance remains unclear (2). This highlights the need for further research to fully understand these variants’ implications.

The variability in clinical presentation among individuals with similar genetic mutations complicates predicting disease severity and outcomes. While our patient experienced multiple EIAKI episodes without high-intensity exercise, others with the same or similar mutations may have been asymptomatic or exhibited mild symptoms. This variability emphasizes the importance of considering the full genetic context, including potential interactions between different variants and other genetic or environmental factors (5).

Comparison with Other Studies: Common themes emerged when comparing cases of compound heterozygous SLC2A9 mutations. Literature shows patients with similar mutations exhibiting a spectrum of symptoms, from asymptomatic hypouricemia to recurrent EIAKI (2). This supports the idea that RHUC is genetically heterogeneous with diverse phenotypes. Previous research identified individuals with the I335N variant of SLC2A9, classified either as pathogenic or VUS based on genetic context and clinical presentation (10, 11). The variability in clinical expression suggests that additional factors—genetic, environmental, or lifestyle-related—significantly influence RHUC manifestation. Comparing our case emphasizes the challenge in establishing clear genotype-phenotype correlations in conditions like RHUC, influenced by multiple factors.

### Clinical implications

The presentation of a 30-year-old Indian male with RHUC illustrates the variability and diagnostic challenges of the condition. Consistent with literature, our patient experienced AKI without an identifiable trigger, a common RHUC pattern often linked to strenuous exercise. However, AKI episodes in this case occurred without high-intensity physical activity, underscoring RHUC’s unpredictable nature and the necessity for heightened clinical vigilance (12).

Clinical Course and Presentation: The patient experienced recurrent AKI episodes that resolved spontaneously, consistent with the natural recovery seen in RHUC-related AKI. This spontaneous recovery highlights the need to recognize this pattern in patients with unexplained kidney injury. Elevated creatine phosphokinase (CPK) without a corresponding increase in lactate dehydrogenase (LDH) suggests muscle stress or mild rhabdomyolysis, indicating muscle damage without significant cell lysis. The elevated indirect bilirubin level may suggest mild hemolysis or Gilbert’s syndrome, characterized by intermittent jaundice. These findings require further investigation to understand their implications for RHUC.

Diagnosis: RHUC was confirmed through clinical history, laboratory tests, and genetic analysis. Low serum uric acid levels, combined with high urinary uric acid excretion, suggested RHUC, confirmed by identifying compound heterozygous mutations in the SLC2A9 gene. Whole-genome sequencing (WGS) was crucial for detecting these mutations, especially in atypical presentations. The identification of the c.646G>A (p.Gly212Arg) and c.1004T>A (p.Ile335Asn) mutations provided significant diagnostic context, with the I335N variant classified as a Variant of Uncertain Significance (VUS) due to its potential impact on splicing and its prevalence in South Asian populations.

Management: The management of RHUC-AKI in this patient centered on supportive care, including ensuring adequate hydration, correcting electrolyte imbalances, and monitoring early signs of kidney injury. Although xanthine oxidase inhibitors like allopurinol were considered to reduce uric acid production, they were not used due to insufficient evidence supporting their effectiveness in RHUC. Instead, the focus shifted to patient education, emphasizing lifestyle modifications such as avoiding high-purine foods, staying well-hydrated, and avoiding strenuous physical activity that could trigger AKI episodes.

A major strength of this case is the comprehensive genetic analysis, which identified compound heterozygous mutations in the SLC2A9 gene. This, combined with thorough clinical evaluations, provided a definitive diagnosis of RHUC2 and offered valuable insights into the pathophysiology of EIAKI in this context. However, limitations include the absence of genetic testing in the patient’s family, restricting the understanding of inheritance patterns. Additionally, as a single case study, these findings may not be generalizable to all patients with RHUC, indicating the need for further research in a larger cohort.

This case adds to the growing literature on RHUC2, particularly regarding EIAKI. While similar cases have been documented, the identification of specific SLC2A9 mutations in this patient provides new insights that align with and expand upon existing research, highlighting the importance of early genetic testing in at-risk individuals.

## Patient perspective

The patient experienced significant relief with a definitive diagnosis of Renal Hypouricemia Type 2 after years of unexplained symptoms and acute kidney injury episodes. The previous uncertainty had caused considerable stress, affecting his physical and psychological well-being. He appreciated the thorough investigation leading to a clear diagnosis and targeted treatment. However, he expressed concerns about lifelong management, especially the necessary exercise routine modifications, given his active lifestyle. Despite these challenges, he reported improved quality of life after adjusting his diet and exercise as recommended by healthcare providers. He remains vigilant about symptom recurrence, stressing the importance of ongoing monitoring and lifestyle adjustments.

## Conclusion

This case report highlights the complexity of Renal Hypouricemia Type 2 (RHUC2) associated with compound heterozygous mutations in the SLC2A9 gene in a 30-year-old Indian male with recurrent exercise-induced acute kidney injury (EIAKI). Significantly, EIAKI episodes occurred even without intense exercise, indicating that factors beyond traditional risk factors can trigger AKI in RHUC2 patients. Although the identified genetic variants were not novel, this case adds valuable insights to the limited RHUC2 literature and underscores the importance of considering this condition in unexplained AKI cases. These findings highlight the need for personalized diagnostic and management strategies in RHUC2 patients.

## Data Availability

The original contributions presented in the study are included in the article/supplementary materials. Further inquiries can be directed to the corresponding author.
